# A Case of Empyema Successfully Treated by Paracentesis Thoracis

**Published:** 1826-07

**Authors:** 


### A CASE OF EMPYEMA SUCCESSFULLY TREATED BY THE OPERATION OF PARACENTESIS THORACIS.—BY THOMAS JOWETT, ESQ. RESIDENT SURGEON TO ST. MARY'S HOSPITAL AND DISPENSARY, NOTTINGHAM.

[*Extra-Limites.*]

ALTHOUGH empyema may be distinguished with considerable certainty in its advanced stages, when circumstances induce us to suspect its existence; it nevertheless sometimes happens that cases do occur, in which the general symptoms are too obscure, even to lead to the necessary examination of the thorax by the eye and percussion. Under such circumstances, the stethoscope will readily furnish important indications; and, as the following case appears to me well calculated to prove the utility of auscultation, I am induced to hope its details will be thought worthy of publication.

Fanny Cockayne, aged nine years and a half, lace-runner, was admitted an out-patient of St. Mary's Hospital and Dispensary, Feb. 24, 1824. At her admission she laboured under pain in the epigastrium, vomiting and other symptoms of gastric irritation or inflammation. The medicines prescribed for her gave relief, and after being once repeated she discontinued her attendance.

On the 23d of March she returned again. Her appearance indicated considerable suffering,—she said she felt ill and weak, but denied having any pain or uneasiness; nor could I elicit a satisfactory opinion as to the nature of her malady, by interrogating my patient or her mother. I prescribed some powders with calomel and rhubarb.

At her next attendance, on Tuesday, March 30, she appeared much worse. It was with great difficulty that she had walked to the dispensary:—the countenance was pale and sallow, and very expressive of pain;—the lips were pallid;—the tongue coated with yellow fur;—the appetite lost;—and the whole body considerably emaciated. So great, indeed, was the unfavorable change that had taken place since her preceding visit, that I was convinced she could not long survive, unless some further insight into the nature of her disease was obtained. With the view to acquire such information, I again closely interrogated my patient and the mother; and I learnt, in addition, that she had *a cough*, attended with a little expectoration,—was feverish in the night,—and had complained for a day or two of a pain in the right axilla, which was not aggravated by taking a deep inspiration.

My next step was to apply the stethoscope, which I did (without removing the clothes) above each of the clavicles, in the angle formed by the bone and the anterior edge of the trapezius muscle. On the *left* side I heard the respiration *very loud:* on the *right* it was *inaudible*.

Having now obtained a clue to the disease, I uncovered the thorax, and noted the following particulars. STETHOSCOPE. On the *right* side of the thorax the *respiration* is no where audible, except in a very slight degree along the spinal column, and in the superior part anteriorly. On the *left* side, it is every where loud. PERCUSSION. The *sound* of the *right* side is *dead* all over; that of the *left* is hollow. MENSURATION. A line drawn horizontally round the thorax, from the ensiform cartilage to the spine, is one inch and a half longer on the *right* side, than on the *left*. To the *eye* the side appears considerably enlarged: the ribs are widely separated from each other; and, instead of being distinguished by their prominency, as in the natural state, they form *depressions* between the intercostal spaces, the integuments of which bulge outwards and yield on pressure with a sensation not unlike fluctuation. The right side is almost passive in the respiratory movements of the thorax, and when the hand is placed upon it, it gives a sensation of smothered heat.

Upon these indications I felt no difficulty in giving the following diagnosis. *Right cavity of the thorax filled with fluid, which compresses the lung.*

(The medicine prescribed contained digitalis and squills.)

Mr. Oldknow, the consulting surgeon of the institution, visited the case the following morning, (Wednesday, March 31,) and having satisfied himself respecting the accuracy of the foregoing diagnosis, resolved to perform the operation for empyema immediately, as it appeared to be the only measure which afforded any prospect of recovery.

At the distance of three inches from the spine, and in the tenth intercostal space, counting from above downwards (that being the place where the bulging of the integuments existed in the greatest degree), he divided, in a line parallel with the ribs, the skin and intercostal muscles, and then continued his incision through the pleura. A stream of pus immediately spouted out to the distance of several feet, and continued to flow for several seconds; a fit of coughing then came on, which caused the stream to jet out forcibly during its action, whilst the inspiration immediately succeeding not only stopped the current, but also sucked air through the wound into the cavity of the thorax.

The whole quantity of fluid drawn off was three pints; more would have flowed, but as she was much exhausted and approaching a state of syncope, it was considered better to close the opening. The pus was of thick consistence,—of a greenish yellow colour,—inodorous,—perfectly uniform,—and did not separate or undergo any change by standing two days exposed to the air.

After the operation the breathing was much easier; she slept several hours in the night without coughing, and was less feverish than before.

Next morning, April 1, the pulse was 110—the surface of the body was warm but not hot—the cough was loose and easy, but occasioned pain at the wound.

By the *stethoscope*, the *respiration* of the affected side was just audible anteriorly as low as the fourth rib, in which extent of space there was *metallic tinkling* on coughing, and a good sound on *percussion.*

*April* 2. Pulse 110—has been much purged.

As she sits up in bed, very feeble *respiration* is audible posteriorly almost to the bottom of the scapula; there is *metallic tinkling* in the same parts as yesterday, when she coughs or inspires deeply. (Some laudanum was prescribed to check the purging.)

*April* 3. Pulse 112, 120—purging abated,—countenance good, and she seems improving,—sleeps well.

*Percussion* gives as good a sound in the anterior superior part of the *right* side, as of the *left;* below the level of the nipple it is more or less *dead.* The *metallic tinkling* is only audible superiorly.

*April* 5. Wound nearly cicatrized—continues to improve. There is some *respiration* as low as the bottom of the scapula; the *metallic tinkling* is not audible, except when she coughs *strongly* as she lies in bed.

*April* 8. Coughs but little,—no expectoration,—no fever,—perspires a little in the night. The *metallic tinkling* disappeared two days ago: the *respiration* is pretty strong above the clavicle, and to the third rib below it; *just audible* as low as the nipple anteriorly, and a little lower in the back: there is *resonance* of the *voice* below the clavicle. *Percussion* gives the same results as before. The side is not enlarged more than one third of an inch in circumference.

*April* 14. The cough is worse, and she expectorates about three ounces of patches of pus mixed with clear mucus, in the course of a day; she sometimes sweats in the night; the posture is mostly on the back, inclining a little to either side, but to the right in preference.

By the *stethoscope* there is *alteration* of the tone of the *voice* below the right clavicle: the *respiration* remains as before. The *impulsion* of the *heart* is perceived in the inferior part of the side,* where the sound on *percussion* is dead.

The resonance and alteration of the voice above the clavicle, induced suspicions of *tubercles in the lungs.*

*April* 16. Pulse very quick,—no night sweats,—expectoration more decidedly purulent and full as abundant.

By the stethoscope, the *alteration* of the *voice* below the clavicle is very evident; I believe it is *pectoriloquism* and not *egophony*, but the difficulty of prevailing with the child to talk prevents my being certain.

*April* 19. Mr. Oldknow visited her yesterday, and agreed with me in thinking the *change of the voice* below the clavicle to be pectoriloquism rather than egophony. The expectoration is diminished, there are no night sweats,—pulse 110.

*April* 21. She is improving again,—no expectoration,—little cough,—pulse 120—slight perspirations the two last nights.

The *resonance* remains as before: the *respiration* is *audible* anteriorly to the level of the nipple, and so low *percussion* gives a *good* sound; lower down, the *sound* is *dead* and the *respiration absent.* The side still appears enlarged.

*April* 24. Pulse 112, 120—cough continues unattended by expectoration—slight night sweats. She complains of pain in the place where the operation was performed, and, on examining, I find there is a painful fluctuating tumonr pointing outwards. (Apply a poultice.)

*April* 26. The tumour burst yesterday, and discharged about two ounces of pale greenish yellow pus, and pus still continues to ooze through the opening in small quantity. Within the last few days, a considerable curvature of the spinal column has taken place. There is *pectoriloquism* and gentle *mucous* rattle below the clavicle: the *respiration* is distinct half way down the scapula.

*April* 28. Pulse 108,—cough rather worse, and there is a slight return of the expectoration. The wound has discharged more freely since the cough increased. (Decoction of cinchona prescribed.)

*May* 1. Pulse 112,—cough the same,—expectoration scanty,—discharge from the wound diminished. Examined with the *stethoscope*, she seems to *cough* through the tube of the cylinder into the ear, when the instrument is applied below the clavicle.

*May* 5. Pulse 108,—no expectoration,—wound sometimes discharges a little pus,—sweats occasionally in the night. The spinal column is more curved, the concavity of the curve being to the right side.

*May* 10. Feels stronger, and has a craving appetite. To the *eye* the right side now appears considerably contracted in all its dimensions; the ribs are drawn very close together; the shoulder is depressed, and the scapula descends lower than on the opposite side. There is a great sinking inwards of the whole of the right side, and a bulging outwards of the left, which appears to be partly occasioned by the curved state of the spine. By the stethoscope, the *respiration* is audible as low as the sixth rib.

*May* 13. Pulse 90, pretty full,—continues improving.

---

* This depended upon the fluid still remaining in the cavity of the chest.

*May* 15. Pulse only 76, and somewhat irregular in strength and frequency: the heart's action is extensive, and felt as much on the right side of the sternum as on the left.

*May* 22. Very little cough and no expectoration—sometimes sweats in the night—wound healed—feels stronger and gains flesh. The spine appears less curved than before. *Respiration* just audible at the bottom of the right side.

*May* 26. The spine is becoming straighter every day. In order to preserve the appearance of the deformity, Mr. Smith, an ingenious artist, has this day made a sketch of the trunk.

*June* 16. She now works part of the day, and is strong enough to walk to the dispensary, a distance of about a furlong. The spine is straighter, the side more expanded, and the *respiration* stronger, although still weak.

*July* 5. The spinal column is become nearly straight, and the right side has re-enlarged, it being only one third of an inch smaller than the left in circumference. The *respiration* is equally strong on both sides, anteriorly, and superiorly; *weak* but distinct in the lower part of the back on the right side. *discharged cured.*

*October* 14. Continues quite well. There is still a perceptible curve in the spinal column: the right scapula descends lower than the left, and the side appears rather the smallest, although there is little difference manifest on measuring. Examined with the stethoscope; the *respiration* is distinctly audible throughout the whole of the right side, but is not so strong every where, as on the left side.

1825. *February* 4. Continues quite well and has grown considerably—she has, however, a slight cough, and generally sleeps on the right side. The contracted state of the side and the spinal column remains the same: the *respiration* is distinct but weak, and there is still a *whispering kind of noise* when she speaks, below the clavicle. Dr. Storer, who had expressed a wish to see the patient, examined the appearance of the thorax a few days ago.

*Remarks.* The researches of modern pathologists have so satisfactorily shewn the majority of thoracic effusions to arise from an inflammatory affection of the pleura, that I am naturally led to consider pleuritis as the most probable cause in this instance. This opinion is rendered more probable by the contraction which took place in the side, and the diminished intensity of the respiration, which continued after the whole of the fluid was absorbed; as these circumstances prove the existence of the false membranes on the pleura, which accompany inflammations of those membranes. It is asserted by authors, that the effusion of pleurisy is more purulent in proportion as the inflammation is more violent; if this be true, it cannot be correct to attribute an inflammatory origin to the present example, as no case could be more truly devoid of an acute character; but I am disposed to doubt the accuracy of the assertion, as it by no means accords with my experience: I have found the effusion much more purulent in one chronic example than in any case of the most acute character which I have examined after death.

The first examination of the thorax was sufficient to remove all doubt as to the nature of this interesting case of disease. The *dead sound* on percussion, and *absence of respiration* throughout the side, were of themselves satisfactory grounds to found the diagnosis upon.

It cannot be denied that the enlarged state of the side, and the fluctuating sensation of the soft parts bulging outwards between the ribs, would most probably have led to the same diagnosis, if the thorax had been exposed to view; but, it must be recollected that, after a careful investigation of the general symptoms, *no motive existed* to induce me to make that examination, and it was not until I had applied the stethoscope that there were any grounds for suspecting the true character of the malady.

Let us now attempt to trace the progress of the case after the performance of the operation. It has been stated, that air was sucked through the wound into the cavity of the pleura, and that the whole of the fluid was not evacuated; it, therefore, follows that the chest, after the operation, contained both *air* and *pus*. The presence of the former was indicated by the *hollow* sound on percussion superiorly, and that of the latter by the *dead* one in the lower parts: whilst the existence of *both* was shewn by the *metallic tinkling*.

At the time when Laennec published his work, he suspected, but was not certain, that the phenomenon of metallic tinkling might exist in cases of empyema and pneumothorax *without* fistulous communication with the bronchia: the passage to which I allude, as well as the relation of the case referred to, is omitted in Dr. Forbes' translation, and I may, therefore, be permitted to quote it. "Il est peut-être un troisième cas dans lequel le tintement métallique peut avoir lieu; mais comme je ne l'ai rencontré qu'une seule fois, et que ce phénomène est nécessairement d'une dureé fort courte, je ne puis guere en parler comme d'une chose certaine, quoiqu'elle m'ait paru évidente. Je venais de reconnâitre, par la comparison de la percussion à l'auscultation, l'existence d'un pneumo-thorax avec empyème, chez un malade dont on trouvera l'observation dans le chapitre suivant.* Je fis mettre le malade sur son séant en continuant à tenir le cylindre appliqué sur sa poitrine et à l'instant j'entendis distinctement un bruit semblable à celui d'une goutte d'eau qu'on laisserait tomber dans une caraffe aux trois quarts vide. Ce bruit était accompagné d'un tintement métallique évident et qui dura près d'une seconde. (De l'Ausc. t. II. p. 106.)

Cockayne's case proves the accuracy of Laennec's suspicion: in it there could not possibly be any illusion:—the phenomenon was distinctly and repeatedly heard for six days; and I, moreover, marked the gradual absorption of the gazeous fluid, by the daily diminution of its intensity, and the progressively decreasing extent of the space in which it was heard.†

During the first fortnight after the operation, no unfavourable symptoms occurred: the absorption of the pus left in the chest was shewn to be going on, by the gradually increasing extent of the respiration. The report of April 14th, gave just ground for new fears: the increased cough—the character of the expectoration—night sweats—and resonance of the voice below

---

* The case alluded to is that of Louis Francois Brouan, page 157, et seq.

† It is due to Dr. Collin, whose useful work, "Des Diverses Méthodes d'Exploration de la Poitrine." (Paris, 1824. 8vo. pp. 116) has more merit than pretension, to state that he has given a clearer description of the metallic sounds and their pathologic indications than is to be found in Laennec's Treatise; and, although his information was most probably derived from Laennec himself, it must be evident to every peruser of the tract, that he has been an *ear-witness* of what he has described.

It may be useful to point out an important, though merely verbal, error in Dr. Forbes' abridged translation of the above work. The passage "in the case of gazeous and liquid effusion at the same time, *and without* bronchial fistula" should be, "*and with* bronchial fistula." See Forbes on the Stethoscope and Percussion, p. 298, and Collin, p. 100.

the clavicle, induced strong suspicion, that the softening of some tubercles existing in the lung was taking place ; and this suspicion was rendered but too probable by the *very evident alteration* of the voice observed on the 16th —the *pectoriloquism* and *mucous rattle* of the 26th, and the appearance of *coughing through the tube* on the 1st of May. The opinion derives further confirmation from the consideration, that if there were tubercles there would most probably be *adhesions* of the top of the lung : and that there were adhesions may be inferred from a circumstance, that scarcely admits of other explanation, viz. the *scarcely audible respiration* in the anterior-superior part of the side, before the performance of the operation.

Upon mature consideration of the whole of the evidence, I am of opinion, that an excavation in the lung was really formed, and it appears probable that it was of a tuberculous character.

The last process to be noticed is the contraction of the side and curvature of the spine, which appear to have commenced about April 24th, and after increasing in degree for several weeks, to have then returned to the natural state. There can be little doubt that the contraction of the side depended upon a tough layer of partly organized lymph, firmly investing the compressed lung and preventing its being sufficiently inflated to fill the vacancy formed, as the fluid was absorbed; and the parietes of the thorax were, consequently, drawn inwards, and the spine curved, so as to diminish the capacity of the cavity.

The subsequent re-enlargement may be explained by the supposition that the layer of the lymph upon the pulmonary pleura, although firm enough to prevent the rapid expansion of the lung, was not so far converted into rigid cellular tissue as to be incapable of gradual distention.*

With respect to the chest enlarging again after having been contracted, a circumstance the possibility of which, does not appear to have been contemplated by any pathologist, I am not aware that there is any other example besides the present on record, except the imperfect notice of a case by Rullier in the Dictionnaire des Sciences Médicales.. Two months and a half after an attack of pleuro-pneumonia, in a girl eleven years of age, a tumour arose in the lower part of the side, and after increasing rapidly, it opened externally, and discharged a large quantity of sero-purulent fluid and flakes of lymph : the patient recovered. "Pendant toute la durée de cette cure naturelle, la poitrine *s'affaissa* beaucoup du côté malade, mais nous avons eu, il y'a peu de jours encore, la satisfaction de constater sur cette jeune personne qui approche de sa seizième année, que cette *difformité qui a diminué* par les progrés de l'âge, n'a plus aujourd'hui rien de choquant"†

Nottingham, 1826.

P. S.—I have twice, very recently, had occasion to resort to paracentesis of the thorax, in hopeless cases of effusion arising from pleurisy : in both instances the operation was the means of *prolonging*, although it did not eventually *save* the lives of the individuals. I have not leisure to give the details of the cases at this time. It may be useful, however, to mention one or two circumstances, which may tend to render the operation generally safer, and even successful in some cases, in which it would be dangerous, without the precaution I am about to relate.

In evacuating fluid from the chest as soon as the great distention of the

* Upon the subject of contraction of the chest the reader may consult Laennec, tome I, p. 369 et seq. or Forbes' Translation, p. 159.

† Tome XII. p. 70. Art. Empyème.

side is removed, *air* is drawn through the opening into the thoracic cavity, during the act of inspiration—especially during the forcible inspirations which precede the act of coughing.

Now, for two reasons, it is desirable to prevent the admission of air into the pleura, because, 1stly, To evacuate *fluid* and to admit *air* in its place is merely to substitute one cause of compression of the lung, for another. 2dly, The admission of air, occasions the decomposition of the fluid remaining in the chest, whereby it is rendered fetid and acrid, and becomes the exciting cause of fresh inflammation of the pleura; independent of the additional compression of the lung, from the gas extricated during the decomposition of the fluid.

For these reasons I propose to perform the operation in the following manner, when any case presents itself in which the removal of thoracic effusion appears desirable.

Having made a small incision through the integuments only, in the place selected for the operation (which will most commonly be in the back in the sixth or seventh intercostal space, but may require varying under some circumstances,) thrust a *small* trocar carefully through the muscles and costal pleura; having withdrawn the trocar and left the canula in the wound, join a tube connected with Reid or Weiss's syringe to the canula, and slowly abstract the fluid by the syringe, continuing to work it so long as the piston moves freely, or, until any symptoms come on which render it necessary to desist. Then remove the canula from the wound, without previously separating it from the syringe, approximate the edges of the integuments by plasters, and apply a compress to make it more secure.

The object I have in view in recommending a *small trocar* to be used, is to make the opening as small as possible, and thereby prevent any danger of the wound re-opening. In both my recent cases, the operation was performed with a scalpel, and the wound was burst open again by coughing some days afterwards. Although the incision appeared to be small at the time, yet, on examining the body in the last case, the costal pleura was found to be perforated to the extent of half an inch.

I am aware that authors recommend a lancet or scalpel to be used, under the idea that there is less danger of wounding the lung with them: it must be evident, however, that a trocar need not be introduced deeper, than any other instrument; besides that the employment of the stethoscope will, in some cases, render it quite safe to thrust a trocar in its whole length into the thorax.

I may add, in conclusion, that the stethoscope and percussion in both instances, furnished the indications of effusion. In neither case was there any dilatation of the side or fluctuation between the ribs. In one case there was a great curvature of the spine, distortion of the whole thorax, and approximation, with immobility, of the ribs.

*Remarks.* The history of the operation of paracentesis thoracis for empyema and hydrothorax, would well deserve an article in a periodical journal, and we shall probably dedicate one to this interesting subject, ere long. Kurt Sprengel has given a most erudite history of this operation, from the days of Hippocrates to the close of the last century, occupying 87 pages of letter-press. In this place we shall only note one curious fact, that the identical improvement suggested by our excellent friend, Mr. Jowett, was actually put in force more than a hundred years ago, and with the best effects. Scultetus affixed to the canula between the ribs a syringe for the purpose of sucking out the pus or other fluid extravasated in the thorax. In the year 1767, Dominique Anel published a work entitled, "L'Art de Succer les Plaies," in which, (Sprengel informs us,) he advocates the operation of paracentesis in hydropic and purulent effusions within the chest, and has described different syringes and other instruments for pumping out the fluids. "Aussi imagina-t-il differentes seringues et autres machines à pomper, avec des canules dont les orifices étaient fort larges et diversement configurés."—*Histoire de Médécine, vol.* 9, *p.* 35. The suction of wounds in the chest followed by extravasation was very common at one period, as we are informed by La Motte, so that when men went out to fight duels, they took suckers with them, and the success of this practice was so great that it was, at length, attributed to the devil, and the priests refused extreme unction to those who submitted to so diabolical a measure! Such were the times in which the "tide of civilisation (as Mr. Canning says,) was rolled back," by intolerant superstition! We think the principle of suction, or, in other words, the knowledge of atmospheric pressure, will soon be very usefully employed in more cases than those of poisoned wounds.—*Ed.*

